# New evidence of bovine leukemia virus circulating in Myanmar cattle through epidemiological and molecular characterization

**DOI:** 10.1371/journal.pone.0229126

**Published:** 2020-02-21

**Authors:** Kyaw Kyaw Moe, Meripet Polat, Liushiqi Borjigin, Ryosuke Matsuura, Si Thu Hein, Hla Hla Moe, Yoko Aida

**Affiliations:** 1 Nakamura Laboratory, Baton Zone Program, RIKEN Cluster for Science, Technology and Innovation Hub, Wako, Saitama, Japan; 2 Department of Pathology and Microbiology, University of Veterinary Science, Yezin, Nay Pyi Taw, Myanmar; 3 Laboratory of Viral Infectious Diseases, Department of Computational Biology and Medical Sciences, Graduate School of Frontier Science, The University of Tokyo, Wako, Saitama, Japan; 4 Department of Anatomy, University of Veterinary Science, Yezin, Nay Pyi Taw, Myanmar; 5 Department of Genetics and Animal Breeding, University of Veterinary Science, Yezin, Nay Pyi Taw, Myanmar; Centers for Disease Control and Prevention, UNITED STATES

## Abstract

Bovine leukemia virus (BLV) is the etiological agent of enzootic bovine leukosis, which is the most common neoplastic disease of cattle. BLV infects cattle worldwide and causes serious problems for the cattle industry. In this study, we examined the prevalence of BLV infection and the distribution of BLV genotypes in cattle in the northern, central, and southern parts of Myanmar. The prevalence of BLV infection among Myanmar cattle (37.04%) in this study was markedly higher than the prevalence (9.1%) observed in our earlier study in which BLV was detected from the limited number of cattle only from a small area of Myanmar. Phylogenetic analysis of partial *env-gp51* sequence of the isolated BLV strains revealed that there are at least three BLV genotypes (genotype-1, genotype-6, and genotype-10) in Myanmar, which have also been detected in the neighboring countries. We performed this study to estimate the BLV proviral load, which is a major diagnosis index for determining the virus transmission risk. The cattle of the three test regions with warm, wet, and humid climatic conditions (upper Sagaing, Yangon, and Kayin) exhibited a high mean proviral load, while cattle of three other regions with low annual rainfall and very high temperature (Mandalay, Magway, and upper Bago) exhibited a low mean proviral load. Further, the level of proviral load and the prevalence of BLV infection in Myanmar native cattle (N = 235) were lower than that in the hybrid cattle (Holstein Friesian × Myanmar native) (N = 62). We also observed that the cattle with high risk for BLV transmission, which have high proviral load, may enhance the BLV infection rate. Hence, to control BLV transmission, it is necessary to eliminate these cattle with high-risk for BLV transmission and to diagnose BLV provirus in cattle in the remaining regions/states of Myanmar sharing a boundary with neighboring countries.

## Introduction

Bovine leukemia virus (BLV) infects cattle worldwide and is the etiologic agent for enzootic bovine leukosis (EBL). BLV belongs to the genus *Deltaretrovirus* of the *Retroviridae* family [1, 2]. BLV is closely related to the human T-cell leukemia virus type 1 and 2 (HTLV-1 and -2) [1]. BLV has also been detected in the human tissues, such as human breast tissue, which suggests a risk for the infection and proliferation of this virus in humans [3].

Majority of the BLV-infected cattle are asymptomatic. However, approximately one-third of the infected animals develop persistent lymphocytosis (PL), which is characterized by the polyclonal proliferation of CD5+ B lymphocytes [1]. Only 1–5% of the BLV-infected cattle exhibit malignant monoclonal B-cell lymphosarcoma. The clinical symptoms of lymphoma, caused by BLV infection, depend on the site of the tumor, but include digestive disturbance, loss of appetite, weight loss, weakness, reduction in milk production or general debility, and various neurological manifestations [4]. In addition, recent studies have reported that BLV infection reduces milk production [5, 6] and is associated with high incidence of infectious disease [7] and reproductive inefficiency, resulting in high culling rates of the BLV-infected but healthy cattle [8]. Hence, BLV eradication is of utmost importance. BLV is transmitted through blood containing infected lymphocytes and through the colostrum and milk. Additionally, farm practices such as tattooing, dehorning, rectal palpation, and injection have also been associated with BLV transmission [1]. Blood-sucking flies are one of the important factors for BLV transmission [9, 10]. BLV infection rates tend to be high in older cattle populations [11].

The BLV genome comprises the essential genes *gag*, *pro*, *pol*, and *env*, which encode structural proteins and enzymes, the regulatory genes *tax* and *rex*, and the accessory genes *R3* and *G4*, and two identical long terminal repeats (LTRs) [1, 12]. Similar to the other members of the *Retroviridae* family, the BLV *env* gene encodes the envelope glycoprotein precursor pr72env, which is important for viral infection and syncytium formation. BLV *env* gene also encodes a mature surface glycoprotein (gp51) and a transmembrane protein (gp30) [13–15]. The glycoprotein gp51 plays a crucial role in the viral life cycle and influences the capacity of BLV to enter cells and has been identified as a target of specific neutralizing antibodies [16–18]. Therefore, the gp51 region has been widely used for BLV genotyping and phylogenetic studies for the identification of viral strains isolated from different geographical regions. Globally, at least 11 different genotypes have been identified [19–36].

Previous studies have demonstrated that the proviral load, which represents the BLV genome integrated into the host genome, is an important index for estimating the BLV infection stage. Proviral load is associated with disease progression [37, 38, 39], lymphocyte count [40], viral biokinetics [41], viral infectivity [42], and virus shedding into the saliva and nasal secretions [43]. Among the cows with high risk for BLV transmission, the cows in which BLV provirus was detected in the nasal secretion and saliva had a proviral load of over 14,000 copies/10^5^ cells and 18,000 copies/10^5^ cells in their blood sample, respectively [43]. It has been suggested that these cows are strongly responsible for BLV transmission in healthy cows via direct contact.

Myanmar, situated in the Southeast Asian mainland, is an agro-based country. Myanmar native cattle are zebu type (*Bos indicus*) and is the main draught animal used in cultivation. They are also used for transportation in the rural area and for meat and milk production [44]. Local breeds of cattle, such as Pyar Sein and Shwe Ni, are widely reared throughout the country. These breeds are adapted to the harsh native environment and are resistant to tropical diseases and external parasites. For the development of local draught cattle, zebu type Shindi and Thari breeds were imported into Myanmar in the 1950s and were crossed with the local breeds. In the 1970s, the local breeds in Myanmar were crossed with the imported dairy cattle breeds such as Holstein Frisian and Jersey to improve their dairy performance. Currently, dairy cattle play a secondary role in ruminant production owing to demand for milk and milk products among the urban populations and improved nutritional standards and quality of the life of the rural populations. In 2017, Myanmar owned 17.14 million heads of cattle (about 1.1% of the world cattle population), based on the FAOSTAT database (http://www.fao.org/faostat/en/#data/QA).

Recently, we have detected BLV infection in different cattle farms of the Nay Pyi Taw Union Territory, Myanmar, and examined the genetic variability in the BLV strains using blood samples of 66 cattle [33]. About 9.1% of the samples were positive for BLV provirus and a phylogenetic tree, constructed using partial and complete *env-gp51* sequences, indicated that Myanmar strains were genotype-10 among the 11 globally detected genotypes. However, only a limited number of samples were tested in the previous study, which were collected from a small and definite area. If samples are collected from different parts of the country, more genotypes might be detected. Moreover, there is a need to examine the diversity of BLV in Myanmar, as three BLV genotypes (genotype-1, genotype-6, and genotype-10) have been recorded in the countries neighboring Myanmar; genotype-1, genotype-6, and genotype-10 in Thailand [32], genotype-6 and genotype-10 in China [45, 46], and genotype-6 in India [36]. In this study, we examined the detailed epidemiological information on the prevalence of BLV in Myanmar using BLV-CoCoMo-quantitative polymerase chain reaction (qPCR) assay [39, 47]. We extracted DNA from 297 bovine blood samples, which were collected from different locations throughout the country. Furthermore, we demonstrated that the level of BLV proviral load was associated with different regional variations and cattle breeds in Myanmar. Additionally, we investigated the genetic diversity of BLV strains detected in Myanmar by phylogenetic analysis of BLV *env-gp51* gene sequence. We detected the presence of at least three BLV genotypes (genotype-1, genotype-6, and genotype-10) in Myanmar.

## Materials and methods

### Animals and extraction of genomic DNA

**Fig 1:**
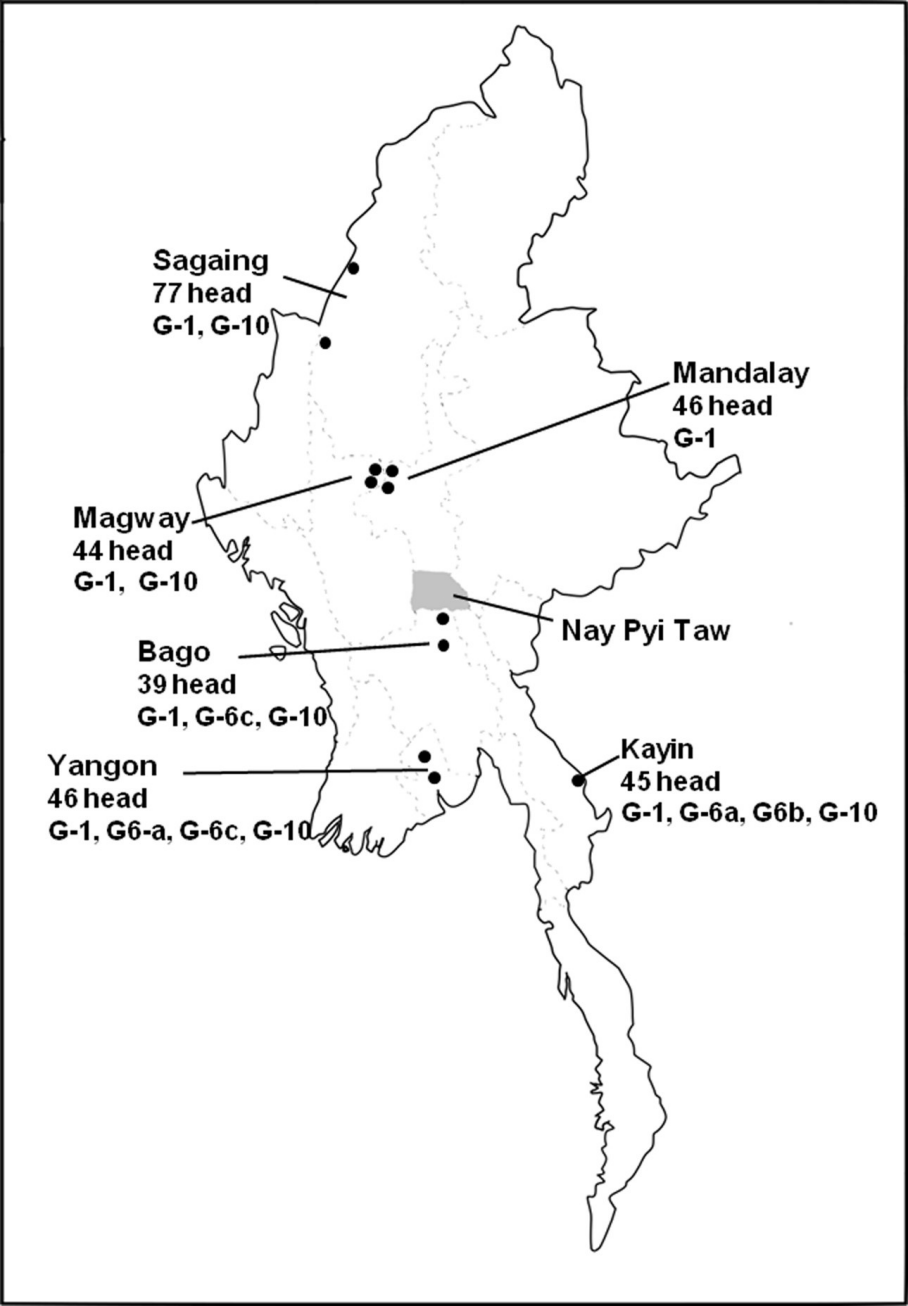
Map of Myanmar showing the number of cattle included in this study and bovine leukemia virus (BLV) genotypes detected in the tested area. Blood sampling was performed in eleven townships (indicated by black color circles) located in one state and five regions of Myanmar. The gray color indicates the blood sampling area in previous study. The figure is created by modifying "map of Myanmar", copyright belongs to (© Keisuke Inoue (Licensed under CC BY 4.0 https://www.freemap.jp/item/asia/myanmar.html).

Blood samples were collected from 297 cattle in 42 different private farms of 11 townships located in 5 regions and 1 state in the northern, central, and southern parts of Myanmar between February and March 2018, as shown in Fig 1. The cattle breeds included native Pyar Sein and Shwe Ni breeds (N = 235), and crossbred Holstein-Friesian breed (N = 62). The age of the cattle ranged from 6-months to 18-years. Most of the farms were small holding farms having less than 10 cattle per farm. The permission from owner was required to access farms and cattle. The genomic DNA was extracted from 200 μL of whole bovine blood using PureLink Genomic DNA Mini Kit (Invitrogen, Carlsbad, CA), following manufacturer’s instructions.

#### Evaluation of BLV provirus by BLV-CoCoMo-qPCR-2

BLV-CoCoMo-qPCR-2 (RIKEN Genesis, Kanagawa, Japan) using THUNDERBIRD Probe qPCR Mix (Toyobo, Tokyo, Japan) was used to determine the presence of BLV and to measure proviral load as described previously [47]. A 183-bp sequence of the BLV LTR was amplified using the degenerate primer pair, CoCoMo-FRW and CoCoMo-REV, and a 15 bp 6-carboxyfluorescein (FAM)-labeled LTR probe. A 151-bp sequence of *BoLA-DRA* (internal control) was amplified using the primer pair DRA-FW and DRA-RW and a FAM-labeled DRA probe as previously described [47]. The proviral load was calculated as follows: (number of BLV LTR copies/number of *BoLA-DRA* copies) x 10^5^ [47].

### PCR amplification of BLV *env*-*gp51* gene fragments and nucleotide sequences

The partial sequence of BLV *env-gp51* gene of the BLV provirus-positive samples was amplified by nested PCR using PrimeSTAR GXL DNA Polymerase (Takara Bio Inc., Otsu, Japan), as described previously [28, 48, 49]. The BLV *env-gp51*-positive PCR products were purified using Exo-SAP IT (USB Corp., Cleveland, OH) and were sequenced on an ABI 3730xl DNA Analyzer using the BigDye Terminator v 3.1 Cycle Sequencing Kit (Applied Biosystems, Foster City, CA). The sequence included a 502 bp region of the *env-gp51* gene, corresponding to the 5078 to 5579 nucleotide position on the BLV whole genome sequence (National Center for Biotechnology Information (NCBI) GenBank accession number K02120) [50]. The nucleotide sequence was edited, aligned, and identified using MEGA 7 software [51].

### Construction of the phylogenetic tree

The partial sequence of BLV *env-gp51* from 66 BLV-positive DNA samples, which were successfully amplified by nested PCR, was aligned with the 44 BLV *env-gp51* sequences from GenBank (representative of the eleven known BLV genotypes) using MEGA 7 software. Phylogenetic analysis of a partial (502 bp) sequence of the *env-gp51* gene was conducted using MEGA 7. For robust and accurate phylogenetic analysis of the BLV *env-gp51* sequence, the ‘‘find best DNA/Protein models” tool of MEGA 7 software was used to choose the best fit model. The Kimura-2 parameter model with gamma distribution (K2+G) was chosen as the model with the best fit to analyze the BLV *env-gp51* sequence with the smallest Akaike information criterion (AIC) values. Two phylogenetic trees were constructed using the maximum likelihood (ML) algorithm with the K2+G model of nucleotide substitution in MEGA 7. The reliability of the phylogenetic relationships was evaluated by nonparametric bootstrap analysis with 1000 replicates. The deduction of protein sequence through translation of nucleotide to amino acid sequence was performed using MEGA 7 [51].

### Statistical analysis

Brunner-Munzel test was used to calculate the significance of the differences of the means of proviral loads between two breeds. Tukey's test after the analysis of variance was used to determine the significance of the means of proviral loads between the different geographical regions. *P*<0.05 was considered significant.

### Animal handling and research ethics

All animals were handled by RIKEN, Japan in strict accordance with good animal practice following the guidelines of RIKEN. The study was approved by the RIKEN Animal Experiments Committee (approval number H29-2-104).

## Results

### Infection rate prevalence of BLV

**Fig 2:**
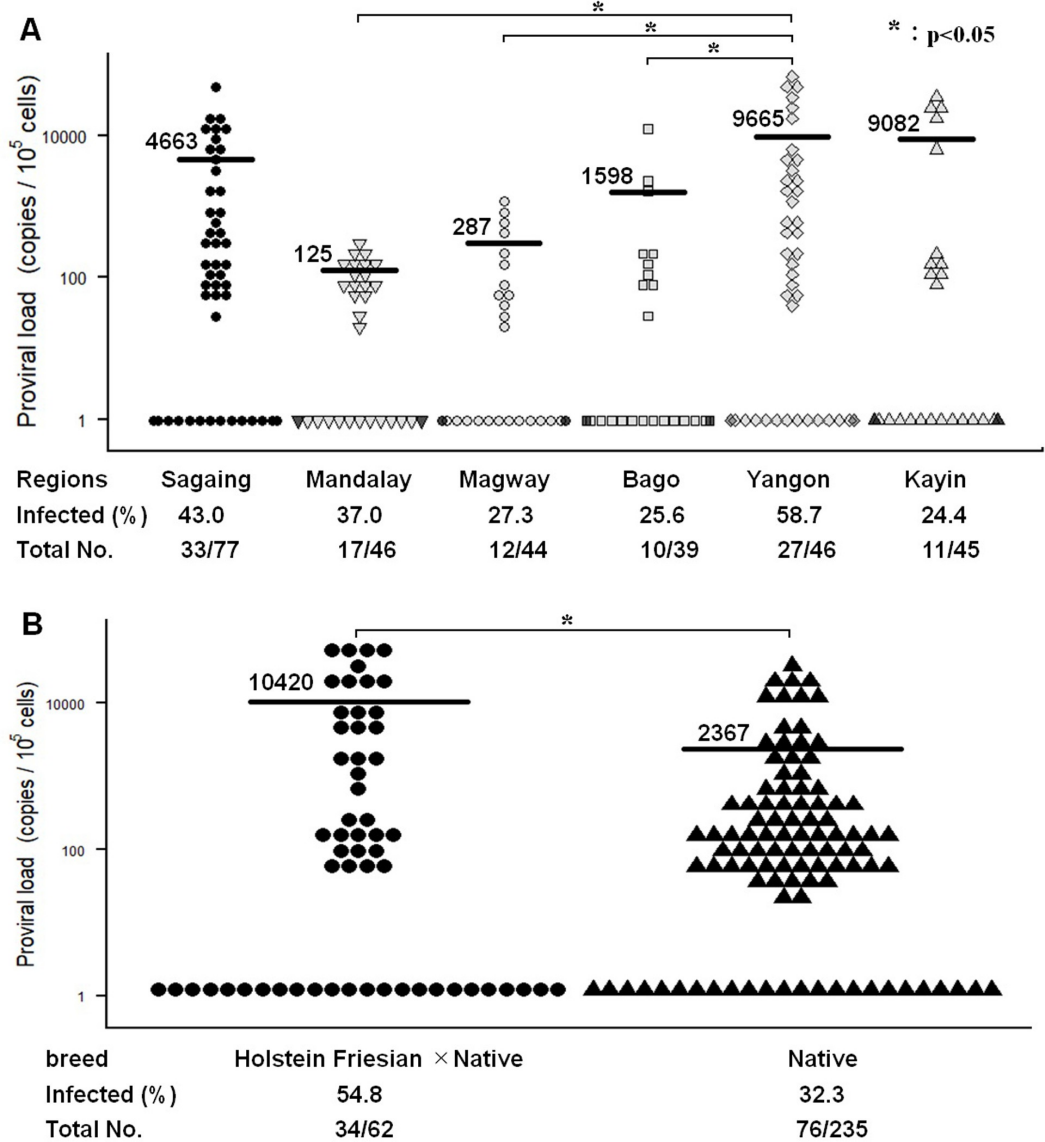
Proviral load and prevalence of bovine leukemia virus (BLV) infection among the cattle of six test areas in Myanmar (A) and among the hybrid (Holstein-Friesian crossbred with native breed) cattle and among the native cattle breed (B). The mean proviral load is indicated for each region/state and each cattle breed. Significant differences between two cattle breeds and between the different geographical regions were calculated by Brunner-Munzel test and Tukey's test, respectively. *P*<0.05 was considered significant.

Previously, we had analyzed BLV infection among the cattle of different farms in the Nay Pyi Taw Union Territory of central Myanmar as highlighted in gray in Fig 1 and we observed an infection rate of 9.1% [33]. In this study, to investigate the prevalence of BLV infection in Myanmar, we collected 297 blood samples of cattle in different farms from 11 townships located in 5 regions and 1 state, which are flanked by Nay Pyi Taw Union Territory (Fig 1). The genomic DNA isolated from these samples was first screened for BLV infection by BLV-CoCoMo-qPCR-2 targeting the BLV LTRs. Surprisingly, BLV infection was detected in all the regions and state tested in this study, in addition to the Nay Pyi Taw Union Territory. Out of a total 297 cattle DNA samples from 42 farms throughout Myanmar, 110 samples (37.04% with a 95% confidence interval extends from 31.74% to 42.66%) from 31 farms were positive for BLV provirus, as determined by BLV-CoCoMo-qPCR-2 (Table 1 and Fig 2A). This indicated that the BLV infection rate was higher than that previously observed in the Nay Pyi Taw Union Territory.

**Table 1:** Summary of bovine leukemia virus (BLV) detection in Myanmar cattle as determined by CoCoMo-quantitative PCR (qPCR).

Region/State	Township	Farm	Breed	Positive % (positive no./tested samples no.)
Sagaing	Tamu	SG1	Native	37.50 (3/8)
			Native × Friesian	66.60 (8/12)
		SG2	Native	17.60 (3/17)
	Kale	SG3	Native	70.00 (7/10)
		SG4	Native	41.60 (5/12)
		SG5	Native	37.50 (3/ 8)
		SG6	Native	40.00 (4/10)
Mandalay	Myingyan	MD1	Native	45.50 (10/22)
	Natogyi	MD2	Native	66.60 (2/ 3)
		MD3	Native	0.00 (0/ 3)
		MD4	Native	22.22 (2/ 9)
		MD5	Native	25.00 (1/ 4)
		MD6	Native	40.00 (2/ 5)
Magway	Yesagyo	MG1	Native	25.00 (5/20)
		MG2	Native	50.00 (1/ 2)
	Pakokku	MG3	Native	100.00 (1/ 1)
		MG4	Native	50.00 (1/ 2)
		MG5	Native	0.00 (0/ 3)
		MG6	Native	0.00 (0/ 1)
		MG7	Native	0.00 (0/ 1)
		MG8	Native	25.00 (1/ 4)
		MG9	Native	100.00 (1/ 1)
		MG10	Native	100.00 (1/ 1)
		MG11	Native	12.50 (1/ 8)
Bago	Yedashe	BG1	Native × Friesian	0.00 (0/ 6)
		BG2	Native	0.00 (0/ 3)
		BG3	Native	0.00 (0/ 1)
		BG4	Native	50.00 (2/ 4)
		BG5	Native	33.33 (2/ 6)
	Taungoo	BG6	Native	50.00 (5/10)
		BG7	Native	20.00 (1/ 5)
		BG8	Native	0.00 (0/ 2)
			Native × Friesian	0.00 (0/ 2)
Yangon	Htantabin	YG1	Native	60.00 (3/ 5)
		YG2	Native	40.00 (2/ 5)
		YG3	Native	75.00 (3/ 4)
		YG4	Native	25.00 (1/ 4)
		YG5	Native	33.33 (1/ 3)
		YG6	Native	0.00 (0/ 3)
	Hmawbi	YG7	Native × Friesian	88.89 (8/ 9)
		YG8	Native × Friesian	69.23 (9/13)
Kayin	Myawaddy	KY1	Native	0.00 (0/15)
		KY2	Native × Friesian	45.00 (9/20)
		KY3	Native	20.00 (2/10)
Total of farms		42	Total	37.04 (110/297)

### Estimation of BLV proviral load

In BLV-CoCoMo-qPCR assay, we amplified a single-copy host gene, *BoLA-DRA* gene to calculate cell number, in parallel with the viral genomic DNA, and calculated the proviral load (expressed as the number of copies of provirus per 100,000 peripheral blood mononuclear cells [PBMCs]). Then we summarized the level of proviral load with BLV infection rate in each tested region/state of Myanmar, as shown in Fig 2A. The BLV-positive farms in two regions (Sagaing and Yangon) and a state (Kayin) exhibited a high mean proviral load (ranging from 4,663 to 9,665 copies), while that in the other three regions (Mandalay, Magway, and Bago) exhibited a low mean proviral load (ranging from 125 to 1,958 copies). Interestingly, Yangon region, where the highest mean proviral load (mean 9665 copies) was observed, exhibited the highest prevalence of BLV infection (58.70%) among the 6 test areas. Particularly, the highest mean proviral load (mean 22,576 copies) was observed among the dairy cattle in farm YG7 located in the Hmawbi Township of Yangon region. The copy number in this farm ranged from 195 to 57,870 copies per 10^5^ cells. Additionally, this farm exhibited the highest infection rate (88.89%) among the 42 farms (Table 1). Seven out of the eight farms tested (87.5%) were positive for BLV provirus in the Yangon region (Table 1). The second highest mean proviral load (9,082 copies) among the 6 test areas was observed in Kayin state. Out of the three cattle farms in Myawaddy Township of Kayin state, the farm KY2 tested positive for BLV provirus (45.0%), with the copy number ranging from 72 to 29,490 copies per 10^5^ cells. The third highest mean proviral load (4,663 copies) among the 6 test areas was observed in Sagaing region. All the six farms in this region tested positive for BLV provirus (100%). The highest mean proviral load (9,000 copies) and the highest prevalence of BLV infection (55.0%) in Sagaing region was observed among the dairy cattle of farm SG1 in Tamu Township with the proviral load ranging from 263 to 44,260 copies per 10^5^ cells (Table 1). In contrast to the cattle of Sagaing, Yangon and Kayin, the rate of BLV-positive farm in Mandalay, Magway, and Bago regions, which had low mean proviral load, was 83.3%, 63.3%, and 66.7%, respectively.

We then compared the prevalence of BLV infection between the native cattle of Myanmar (Pyar Sein and Shwe Ni) (N = 62) and the hybrid cattle (Holstein Friesian × Myanmar native) (N = 235) (Fig 2B). The BLV infection prevalence among the hybrid cattle was 54.84% (34 out of 62 heads), with the copy number ranging from 46 to 57,870 copies per 10^5^ cells (mean 10,420 copies). The prevalence of BLV infection among Myanmar native cattle was 32.34% (76 out of 235 heads), with the copy number ranging from 21 to 36,467 copies per 10^5^ cells (mean 2,367 copies) (Fig 2B). These results demonstrated that the prevalence of BLV infection and the level of proviral load among Myanmar native cattle tend to be significantly lower than those observed among the hybrid cattle. Nonetheless, the difference in sample size is quite large (62 vs 235), and the hybrid cattle is unevenly distributed among 6 different geographical regions, additional studies with more animals each geographical region are required to further confirm these findings.

### Phylogenetic analysis of the partial sequence of BLV *env-gp51*

A previous phylogenetic analysis based on partial and complete *env-gp51* sequence of the BLV strains isolated from only the Nay Pyi Taw Union Territory revealed that the presence of BLV genotype-10 in Myanmar [33]. In this study, to gain further insight into the degree of genetic variability among the BLV strains in Myanmar, phylogenetic characterization was carried out after sequencing the *env-gp51* gene. We selected 83 out of the 110 BLV-positive DNA samples as determined by BLV-CoCoMo-qPCR-2, 66 samples were successfully amplified for the BLV *env-gp51* gene using nested PCR (Table 1). This result showed the same tendency as discussed in our previous publications [28, 39, 52], indicating that BLV-CoCoMo-qPCR-2 has better sensitivity as compared with nested PCR. Indeed, Table 2 shows that proviral load of positive cows (N = 66), as determined by nested PCR for BLV *env-gp51* gene, were detected at a significantly higher level as compared with that of cows negative for provirus (N = 17) as determined by with nested PCR. Then the 66 DNA samples were sequenced and subjected to phylogenetic analysis. The 502 bp sequence corresponding to the nucleotide positions 5,078 to 5,579 of the full-length BLV genome was aligned with the 44 nucleotide sequences from known BLV strains representing all existing BLV genotypes (genotype-1 to genotype-11). The similarity of the 502 bp *env-gp51* nucleotide sequence ranged from 95.2 to 100% among the 66 Myanmar BLV strains used in this study (S1 Table). The degree of similarity of the nucleotide sequence with the 44 nucleotide sequences of 11 known genotypes deposited in GenBank ranged from 92.9 to 100% (S2 Table).

**Table 2:** Association between proviral load as determined by BLV-CoCoMo-qPCR-2 and BLV infection rate as determined by nested PCR for BLV *env-gp51* gene [*p* Value = 0.000454, odds ratio (95%CI) = 7.125 (2.18–23.3)].

		Nested PCR	
		Positive	Negative
CoCoMo-qPCR	High (>100/10^5^cells)	57	8
	Low (≤100/10^5^cells)	9	9

**Fig 3:**
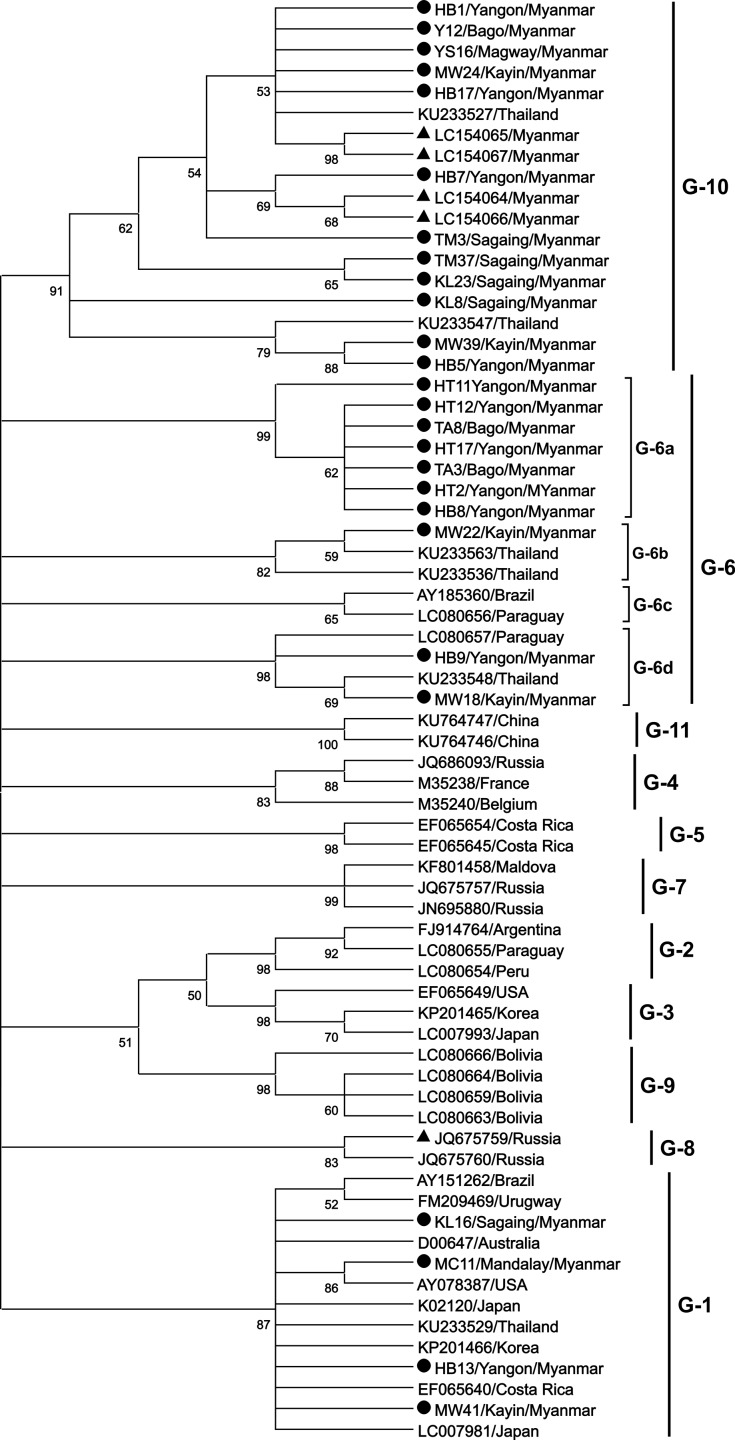
Maximum likelihood phylogenetic tree based on 502 bp nucleotide sequence of the *env-gp51* gene from 66 Myanmar bovine leukemia virus (BLV) strains and 44 known BLV strains isolated elsewhere. Myanmar BLV strains are indicated by the sample ID along with the region/state name and country name. The remaining isolates in the phylogenetic tree are indicated by accession number and country of origin. Distinctive sequences of Myanmar BLV strains are indicated by filled (λ) circles. The 26 typical BLV isolates (accession numbers LC466589–LC466614) were aligned as shown in Fig 5A and indicated by filled (λ) circles. Myanmar BLV strains isolated in our previous study are indicated by filled triangles (▲). The genotypes are indicated by the number towards the right of the figure.

We constructed a ML phylogenetic tree using the K2+G model of nucleotide substitution [51], as shown in Fig 3. The results of phylogenetic analysis were similar to those published in the previous studies and BLV strains were classified into 10 genotypes. The 66 Myanmar BLV strains isolated in this study showed high homology that 26 distinctive sequences of Myanmar BLV strains were used in the phylogenetic analysis. Among these distinctive sequences 4 distinctive sequences from 23 strains clustered with genotype-1. The degree of similarity of these sequences ranged from 99 to 100% when compared with the 9 nucleotide sequences of the BLV genotype-1 (S3 Table). Further, the 10 distinctive sequences of the 16 BLV strains clustered with that of genotype-6 and were 95.7 to 100% similar to the 7 nucleotide sequences of the genotype-6 (S4 Table). The 12 distinctive sequences of the remaining 27 strains clustered with that of the genotype-10 and were 97.7 to 99.8% similar to 6 nucleotide sequence of the genotype-10 (S5 Table).

**Fig 4:**
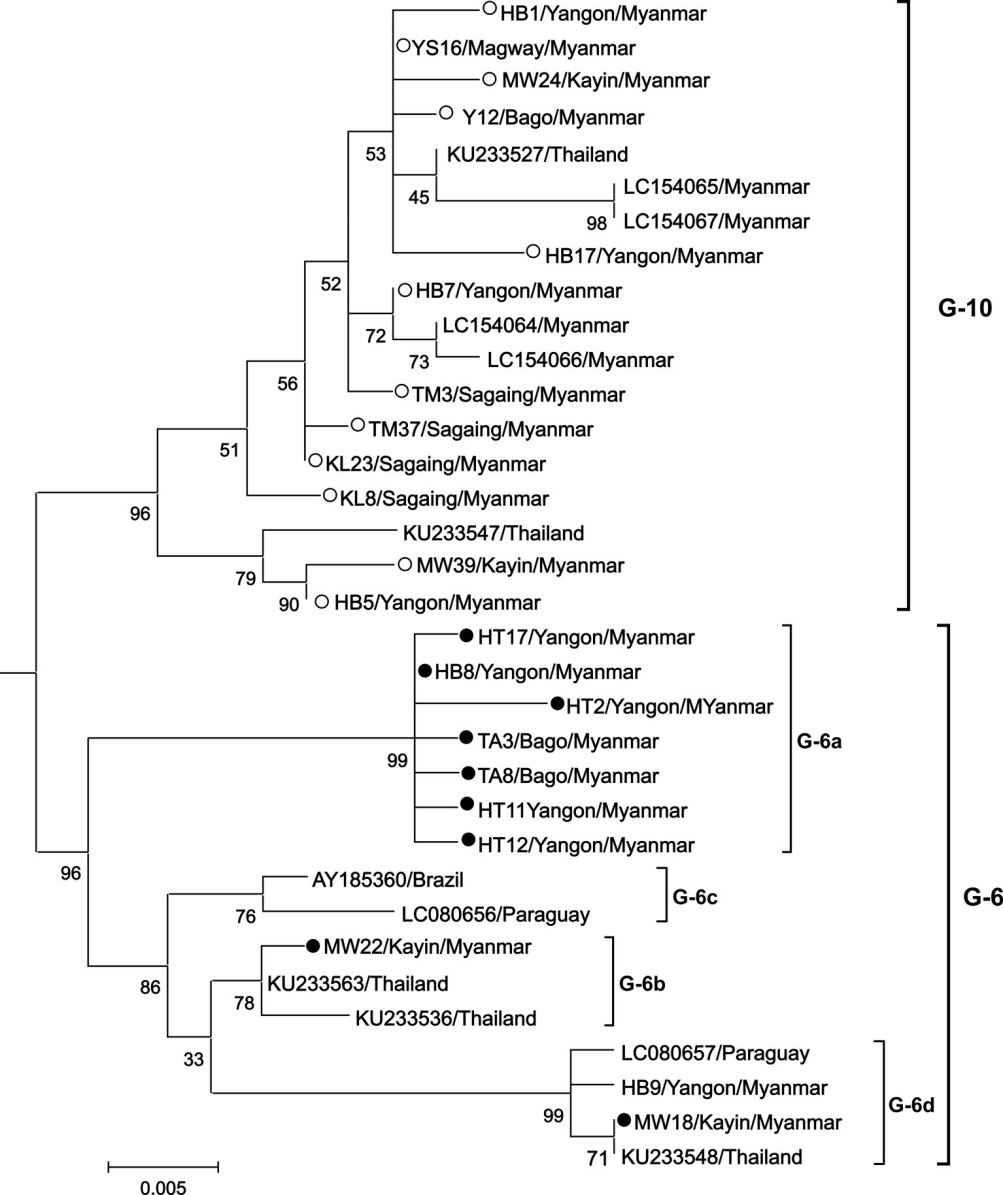
Maximum likelihood phylogenetic tree based on 502 bp nucleotide sequence of the *env-gp51* gene of Myanmar bovine leukemia virus (BLV) strains and other strains representing genotype-6 and genotype-10. Myanmar BLV strains isolated in this study are indicated by the sample ID along with the region/state name and country name. The remaining isolates in the phylogenetic tree are indicated by the accession number and country of origin. Myanmar BLV strains in genotype-6 and genotype-10 are indicated by filled dark circles (●) and open circles (○). The genotypes are indicated by the number towards the right of the figure. The bar at the bottom of the figure denotes the distance.

Phylogenetic analysis indicated four separate subgroups within the genotype-6: G-6a, G-6b, G-6c and G-6d (Fig 3). To confirm the presence of 4 subgroups of genotype-6, a separate ML phylogenetic tree was constructed with the sequences of Myanmar genotype-6 and genotype-10 strains together with other reference sequences of genotype-6, and genotype-10 worldwide (Fig 4). As shown in Fig 4 the sequence of Genotype-6 clustered into 6 subgroups. This subgrouping is supported by bootstrap value ranging from 76% to 99% (Fig 4) and the inter-subgenotype pairwise distance value ranging from 2.3% to 4.3% (S6 Table).

### BLV genotype distribution in Myanmar

Phylogenetic analysis revealed that there are 3 BLV genotypes in Myanmar (Figs 3 and 4), as summarized in Fig 1. BLV genotype-1 was the most prevalent genotype and was detected in all of the six tested areas of Myanmar. The genotype-10 was detected in 5 tested areas except Mandalay region. The genotype-6 was detected in the 3 regions of southern Myanmar (G6a in Yangon and Kayin, G6b in Kayin and, G6c in Bago and Yangon).

### Nucleotide and amino acid substitution in the BLV *env* gp51 gene of BLV strains isolated in Myanmar

**Fig 5:**
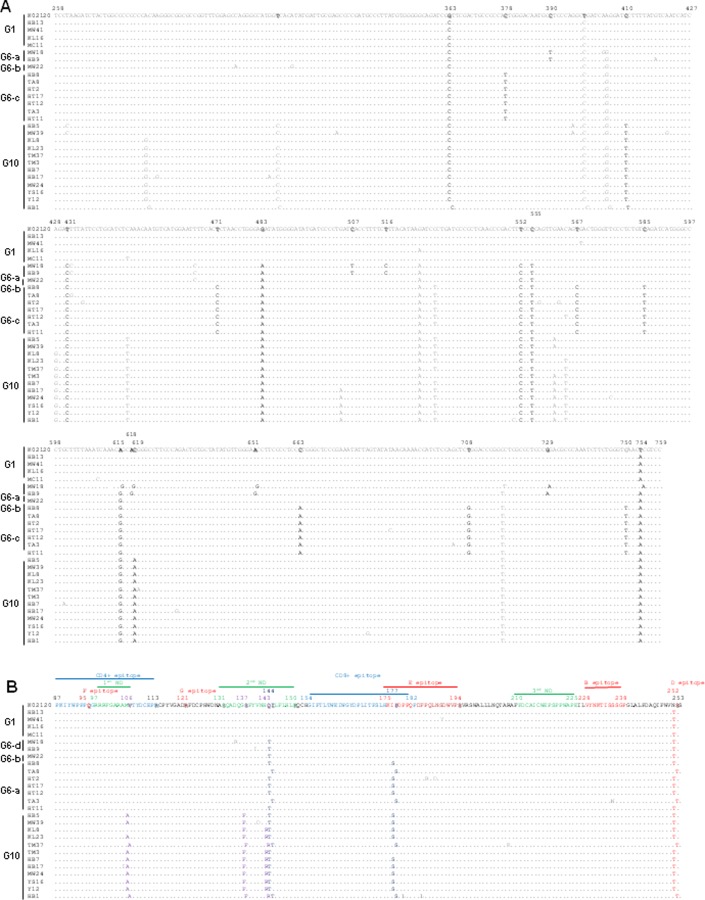
Alignment of the nucleotide and deduced amino acid sequences of the partial bovine leukemia virus (BLV) *env-gp51* gene. Nucleotide sequence (A) and deduced amino acid sequence (B) of the twenty six Myanmar BLV strains were aligned with the reference sequence of genotype-1 (Japanese K02120 strain: GenBank accession number K02120). The Myanmar BLV strains identified in this study are indicated by sample ID. The genotypes are indicated by the vertical lines towards the far left of the figure. The number on the top of the nucleotide sequences indicate the first and last nucleotide positions, and the position of nucleotide substitutions. The number above the deduced amino acid sequence is the amino acid residue number that indicate the start and end of each domain and the position of amino acid substitution. The first, second, and third neutralizing domains (ND) as well as other epitopes are shown at the top of the alignment. Dots indicate identity with the reference BLV strain K02120.

One typical sequence among those that showed 100% identity was submitted to GenBank (accession numbers LC466589–LC466614). Twenty six selected nucleotide sequences from the 66 Myanmar BLV strains were aligned with that of the Japanese K02120 strain (GenBank accession number K02120), a reference sequence (Fig 5A). All of these are indicated by filled circles in the ML phylogenetic trees (Figs 3 and 4).

The nucleotide sequence of all BLV strains that clustered in genotype-1 are mostly similar to that of the reference strain K02120. Myanmar BLV genotype-1, genotype-6 and genotype-10 exhibited two common silent substitutions at nucleotide (nt) 363 and nt 399, corresponding to amino acid residues 121 and 133, respectively. In contrast, Myanmar BLV strains in the three subgenotypes of genotype-6 exhibited diversity in their nucleotide sequence (Fig 5A). Two strains, MW 18 and HB9 in the BLV subtype 6a, exhibited six unique silent substitutions of nt 390, 507, 516, 618, 651, 243, corresponding to amino acid residues 130, 169, 172, 206, 217 and 243, respectively. The remained strains of subtype 6c exhibited seven unique silent substitutions at nt 378, 471, 567, 585, 663, 702 and 750, corresponding to amino acid residues 126, 157, 189, 195, 221, 236 and 250.

All or most of the Myanmar BLV strains belonging to genotype-6 and genotype-10 exhibited six common silent substitutions in the third base of the residues 135, 161, 184, 185, 205, and 236, which were not affected by the variations in nt 405, 483, 552, 555, 615 and 717, respectively. All of the BLV strains that clustered in the genotype-10 exhibited a unique silent substitution in the third base of the residue 207 (nucleotide 619) (Fig 5A).

Most of the silent nucleotide substitution of Myanmar BLV strains were located within the epitopes, including the viral G epitope (nucleotide 363), the second neutralizing domain (ND) (nucleotides 390, 399, 405, and 447), the CD8+ T-cell epitope (nucleotides 471, 483, 507 and 516), the E epitope (nucleotides 552, 555 and 567), the third neutralizing domain (ND) (nucleotides 651 and 663), and the B epitope (nucleotide 708) as shown in Fig 5B.

Twenty-six deduced amino acid sequences representing the sixty-six different Myanmar BLV strains were aligned with the predicted amino acid sequence of K02120. Fig 5B shows the distribution of amino acid changes within the region of *env-gp51* (amino acid positions 87 to 253), which includes a portion of the first ND (residues 97–106), second ND (residues 131–150) and third ND (residues 210–225), a portion of the CD4+ T-cell epitope (residues 87–113) and CD8+ T-cell epitope (residues 154–182), and the viral F (residues 95), G (residues 121), E (residues 175–194), B (residues 228–238) and D (residues 251–253) epitopes [26, 53]. Myanmar BLV strains exhibited highly conserved regions in the predicted amino acid sequences of the partial *env-gp51* gene. The deduced amino acid sequence of the Myanmar genotype-1 strains exhibited high homology to that of the Japanese K02120 strain. Six amino acid variations were detected among the Myanmar BLV strains. Three amino acid substitutions were specifically detected in all or most of the genotype-10 strains. They were substitution of V106A within the CD4+ T-cell epitope and the first ND, substitution of S137Q and substitution of Q143R within the second ND. Furthermore, the genotype-6 strains and genotype-10 strains exhibited a common amino acid substitution of I144T within the second ND and the genotype-6c strains and genotype-10 strains exhibited amino acid substitution of P177S within a portion of CD8+T-cell epitope and the E epitope. All of the Myanmar strains of the three genotypes exhibited substitution of S252T within the D epitope (Fig 5B).

## Discussion

This study focused on the epidemiologic and molecular characterization of BLV strains in Myanmar and leads to three major conclusions. Firstly, we used BLV-CoCoMo-qPCR targeting BLV LTRs to determine the BLV infection rate among cattle in Myanmar. The data clearly indicated that the prevalence of BLV infection among Myanmar cattle was 37.04% with a 95% confidence interval extends from 31.74% to 42.66%, which is markedly higher than the prevalence of BLV infection (9.1%) reported in our earlier study. The low prevalence detected in our earlier study may be because BLV was detected from a limited number of DNA samples collected only from a small area of Myanmar [33]. Secondly, we, for the first time in Myanmar, successfully distinguished the cattle with a high proviral load from those with a low proviral load. This indicated that there was a correlation between the level of BLV proviral load and the BLV infection rate, geographic region, and cattle breeds. Third, the phylogenetic analysis of the partial *env-gp51* gene sequence of the BLV strains isolated in Myanmar revealed that there were three genotypes, genotype-1, genotype-6 and genotype-10 of BLV in the country. The results of this study are more accurate than that from our earlier study on BLV strains isolated from Nay Pyi Taw Union Territory, which detected only genotype-10 in Myanmar [33]. This study also demonstrated that the three BLV genotypes, genotype-1, genotype-6 and genotype-10, were similar to those identified in the neighboring countries of Myanmar [32, 36, 45, 46]. Interestingly, the BLV strains belonging to genotype-6 clustered into 3 subgenotypes and the BLV strains belong to the subtype-6a had distinct nucleotide and amino acid substitutions.

Previously, we had shown an infection rate of 9.1% using cattle from several farms in the Nay Pyi Taw Union Territory of central Myanmar [33]. In this study, we investigated the prevalence of BLV in 11 townships in five regions and 1 state located in the northern, central and southern parts of Myanmar. We demonstrated that the BLV infection prevalence was 37.04% (110 out of 297 head). When we compared the BLV infection rate among the 6 test areas, we observed that the highest infection rate was in Yangon region (58.70%) and the lowest was in Kayin State (24.44%). The BLV infection rate among the cattle of Myanmar reported in this study was higher than that reported in some Asian countries: Philippines (4.8–9.7%) [28] and India (27.9%) [36]. However, the BLV infection rate in this study was close to or lower than that in Korea (42.16%) [29], Thailand (58.74%) [32], and China (49.11%) [46].

BLV proviral load is a major diagnosis index for determining the virus transmission risk. To the best of our knowledge, this is the first study to estimate the proviral load among Myanmar cattle. We observed that the levels of BLV proviral load vary by geographical region within Myanmar. BLV-positive cattle in the three test areas (Sagaing, Yangon, and Kayin) exhibited high mean proviral load, while 3 other test areas (Mandalay, Magway and Bago) exhibited low mean proviral load. Furthermore, we demonstrated that the level of mean proviral load in the BLV-positive dairy farms correlated with the BLV infection rate in the dairy farms. Yangon region, which exhibited the highest mean proviral load also exhibited the highest prevalence of BLV infection among the 6 test areas. Additionally, dairy cattle in the farm YG7 located in Hmawbi township of Yangon region exhibited the highest mean proviral load and the highest infection rate (88.89%) among the 42 farms in the region. Our previous report [43] demonstrated that the cows with a proviral load of over 14,000 copies/10^5^ cells secreted BLV provirus into the nasal secretion. It has been suggested that the cows with the proviral load over 10,000/10^5^ cells have a higher risk of BLV transmission, while those with the proviral load under 10,000/10^5^ cells have a lower risk of BLV transmission. In our study we found that the three regions, which had high mean proviral load, were also associated with the high risk for BLV transmission (6, 6, and 4 for heads for Sagaing, Yangon and Kayin, respectively) as compared with that in the three regions which showed low mean proviral load (0, 0, and 1 heads for Mandalay, Magway and Bago, respectively) (Fig 2A). Similarly, the presence of cows with high risk for BLV transmission among the hybrids (Holstein Friesian × Myanmar native) (9 heads) was higher compared to that among the Myanmar native herds (8 heads) (Fig 2B). Thus, our observation indicates that depending on the geographical region and the population of cows with high risk for BLV transmission (proviral load over 10,000/10^5^ cells) may increase the BLV infection rate.

The climate of Yangon region and Kayin state, situated in southern Myanmar, is warm and is associated with high annual rainfall and humidity. Two sampling townships (Tamu and Kale) in the northern part of Sagaing region are geographically hilly and receive high annual rainfall. High rainfall cause floods in these area with long lasting wet condition. The climatic conditions may be one possible factor for the difference in BLV infection prevalence. Warm, wet, and humid climatic conditions in Sagaing, Yangon and Kayin may favor the blood sucking flies to transmit BLV from the infected animal to the healthy animal in the cattle farms, which result in higher proviral load and infection rate. Blood-sucking flies are regarded as one of the important mediators of BLV transmission [9, 10]. Interestingly, Mandalay and Magway regions, which are located in the central dry zone of Myanmar, and the sampling area in northern part of Bago region, which is closer to the central dry zone, have low annual rainfall and very high temperature (above 40°C in summer). Hence, these regions may be inhospitable for the survival of blood sucking insects.

The phylogenetic analysis based on partial and complete *env*-*gp51* gene sequence in our previous study indicated that the isolated Myanmar strains were of genotype-10 [33]. The limited number of samples tested in the previous study, which was collected only from a small area of Myanmar, may be the reason for isolating only a single genotype. Hence, we hypothesized that if the samples are collected from different regions of the country, more genotypes of BLV may be identified. Indeed, three BLV genotypes (genotype-1, genotype-6 and genotype-10) were detected in our study based on the phylogenetic analysis of the partial *env*-*gp51* nucleotide sequence of the BLV strains isolated from cattle reared in different regions of the country. Genotype-1 was the most prevalent genotype and was detected in the six test areas, while genotype-10 was detected in five test areas and genotype-6 was detected in only three test areas of Myanmar, as shown in Fig 1. Interestingly, the presence of two genotypes in the same herd was detected in some cattle farms of Sagaing, Bago, Yangon regions and Kayin state.

It is interesting to determine the origin of 3 different BLV genotypes discovered in this study. Myanmar has a history of importing exotic cattle breeds. Holstein Frisian and Jersey from Australia and New Zealand were imported in the 1970s for the artificial insemination project [44] and BLV genotype-1 seems to be existing for many years ago together with foreign cattle importation. The BLV genotype-6 has been detected in Brazil [23], Argentina [54], Peru, Paraguay and, Bolivia [31] in South America and Philippines [28], Thailand [32], India [36], and China [46] in Asia. Shindi and Thari breeds were imported to Myanmar many decades ago for the development of local draught cattle [44], but these cattle breeds came from Pakistan and not from India. The recently reported BLV infected regions in India [36] and China [46] are very far from the southern part of Myanmar where BLV genotype-6 was detected. Although the Philippines is situated in Southeast Asia, it is also very far from Myanmar and there is no cattle trade between the two countries. Based on these facts, it is unlikely that the origin of Myanmar BLV genotype-6 is India, China, or the Philippines. BLV genotype-1, genotype-6, and genotype-10 have also been reported in Thailand [32]. Furthermore, our phylogenetic analysis also indicated that the Myanmar BLV genotype-6 subtypes, G6a and G6b strains, exhibited homology to Thailand genotype-6 strains but not to Brazilian or Indian genotype-6 isolates. Myanmar genotype-10 strains isolated in our previous study and in this study were closely related to the genotype-10 strains of Thailand (Figs 3 and 4). Although there is no official information on live cattle trade between Myanmar and Thailand, there is a report by World Food and Agriculture Organization (FAO) that Thailand exported frozen semen of Brahman cattle and dairy cattle to its neighboring countries including Myanmar [55]. Recently, dairy and beef cattle have been imported into Myanmar through border trade. Therefore, we could speculate that the BLV strains, particularly genotype-6 and genotype-10, may have been transmitted to Myanmar through live cattle movement or cattle imported from Thailand.

Several studies have reported the existence of subgroups within BLV genotype-6, based on partial and/or complete *env*-*gp51* sequence [28, 31, 32, 36]. We also reported an important finding of additional subgenotypes within BLV genotype-6 in this study. The topology of the phylogenetic tree in Fig 4 clearly elucidates the clustering of genotype-6 and its 3 subgroups. This finding was supported not only by the phylogenetic analysis of *env*-*gp51* partial gene sequence with significant branch support (bootstrap values) and pairwise distance values, but also by the unique nucleotide substitutions. Interestingly, most of the Myanmar BLV strains in G6 clustered as subgroup 6c separately. The distinct features of the G6c strain were unique silent nucleotide substitutions at seven positions: 378, 471, 567, 585, 663, 708, and 750, and an amino acid substitution namely proline to serine at residue 177 within a portion of CD8+T-cell epitope and the E epitope, which were not observed in the other G6 strains isolated elsewhere (Fig 5A and 5B).

Most of the nucleotide and amino acid substitutions of Myanmar BLV genotype-6 and genotype-10 strains were located within the epitopes, including CD4+ T-cell epitope, the first ND, the viral G epitope, the second ND, the CD8+ T-cell epitope, the E epitope, the third ND and the B epitope (Fig 5A and 5B). These results were consistent with previous reports on the BLV genotype-6 and genotype-10 strains isolated elsewhere [28, 31, 32, 36, 54].

The present study indicated that the BLV prevalence (37.04%) is relatively higher and three BLV genotypes (genotype-1, genotype-6 and genotype-10) are commonly detected in Myanmar cattle than those observed in our previous study (9.1% prevalence and only genotype-10). BLV may have been transmitted to Myanmar through live cattle movement or cattle import, as these 3 BLV genotypes have also been detected in the neighboring countries. The introduction of infected cattle such as dairy breeds is considered as a major driver for BLV infection. To establish an appropriate cattle management policy for the control of BLV infection in Myanmar, it is necessary to issue a regulation to eliminate the cattle with high risk for BLV transmission, which exhibit a proviral load of over 10,000/10^5^ cells, and to allow rearing of only the cattle with low risk for BLV transmission, which exhibit a proviral load of under 10,000/10^5^ cells. Hence, there is a need to identify and characterize BLV strains among the cattle in the 7 out of 8 remaining regions/states of Myanmar which have boundaries with the neighboring countries to prevent BLV transmission into the country.

## Supporting information

S1 Table Evolutionary divergence among the nucleotide sequence of 66 Myanmar bovine leukemia virus (BLV) strains isolated in this study. (XLSX)

S2 Table Evolutionary divergence between the nucleotide sequence of 66 Myanmar bovine leukemia virus (BLV) strains and 40 known strains of 10 genotypes used for the phylogenetic analysis. (XLS)

S3 Table Evolutionary divergence of nucleotide sequence between the 23 Myanmar bovine leukemia virus (BLV) genotype-1 strains and 10 known strains of genotype-1 used for the phylogenetic analysis. (XLS)

S4 Table Evolutionary divergence of nucleotide sequence between the 16 Myanmar bovine leukemia virus (BLV) genotype-6 strains and 7 known strains of genotype-6 used in the phylogenetic analysis. (XLS)

S5 Table Evolutionary divergence of nucleotide sequence between the 26 Myanmar bovine leukemia virus (BLV) genotype-10 strains and 6 known strains of genotype-10 used in the phylogenetic analysis. (XLS)

S6 Table Inter-subgenotype pairwise evolutionary divergence among the 3 subgenotypes of Myanmar genotype-6 strain. (XLS)
